# Infection Incidence and Survival in Patients With Multiple Myeloma and Chronic Lymphocytic Leukaemia: A Growing Concern in the Era of Modern Therapies

**DOI:** 10.1002/jha2.70363

**Published:** 2026-07-31

**Authors:** Ingrid Glimelius, Love Tätting, Jonatan Freilich, M. Natalia Stelmaszuk, Qian Yang, Anna Deleskog, Sigurður Y. Kristinsson

**Affiliations:** ^1^ Department of Immunology, Genetics and Pathology, Cancer Precision Medicine Uppsala University Uppsala Sweden; ^2^ Blood Oncology and Tumour Disease Oncology Clinic Akademiska University Hospital Uppsala Sweden; ^3^ Department of Haematology in Linköping and Department of Biomedical and Clinical Sciences Linköping University Linköping Sweden; ^4^ Access Consulting – RWE Parexel International Stockholm Sweden; ^5^ Department of Public Health and Clinical Medicine Umeå University Umeå Sweden; ^6^ RWE Consulting – Epidemiology & COA Parexel International Stockholm Sweden; ^7^ Takeda Pharma AB Stockholm Sweden; ^8^ Faculty of Medicine University of Iceland Reykjavik Iceland

**Keywords:** chronic lymphocytic leukaemia, multiple myeloma, immunodeficiency, infections

## Abstract

**Introduction:**

Patients with multiple myeloma (MM) and chronic lymphocytic leukaemia (CLL) are burdened by immunodeficiency due to treatment and underlying disease. Infection‐related mortality remains a significant concern for patients' overall survival (OS).

**Methods:**

This Swedish, population‐based study examined incidence of infections and OS in patients with MM or CLL between January 2010 and December 2021, using Swedish Cancer Registry data.

**Results:**

Overall, 15,766 patients were included (MM: 8532; CLL: 7234); the majority were male (MM: 57%; CLL: 62%); median age was 71 years (range: 19–100 [MM]; 23–102 [CLL]). The proportion with a Charlson Comorbidity Index ≥ 5 was 37% (MM) and 25% (CLL). At least one infection was reported in 66% (MM; *n* = 5618) and 50% (CLL; *n* = 3618), with a median follow‐up of 3.3 and 4.8 years, respectively. Pneumonia was the most common bacterial infection (21% for both), while herpes zoster was the most common specified viral infection (MM: 2.6%; CLL: 3.5%). Median OS (95% confidence interval) among these patients was 4.7 (4.6–4.9) and 9.7 (9.3–10.2) years, respectively. Infection (any) was the underlying/contributing cause of death for 1251/4822 (26%) deceased patients (MM) and 835/2571 (33%) (CLL). Higher comorbidity, prior infection and prophylaxis were associated with increased infection risk, while age, female sex and more recent diagnosis were associated with reduced risk.

**Conclusions:**

This study demonstrates high mortality rates among patients with MM and CLL, due to underlying malignancy and high infection burden. This emphasises the need for optimised anticancer treatments and infection control strategies.

**Trial Registration:**

The authors have confirmed clinical trial registration is not needed for this submission.

## 1 Introduction

Multiple myeloma (MM) and chronic lymphocytic leukaemia (CLL) are haematological malignancies characterised by disease‐ and treatment‐induced immunodeficiency [1, 2, 3, 4] and need for prolonged and intensive treatment regimens [5, 6]. In 2020, the estimated age‐standardised incidence for MM in Northern Europe was 3.8/100,000 individuals [7]; in 2022, for CLL in Central Europe, this was 3.0/100,000 individuals [8].

Despite significant therapeutic advances in MM/CLL [7, 9], infections remain a major cause of mortality, with a fatal outcome occurring in ∼20% of patients with MM [1] and 25%–50% of those with CLL [10]. Secondary immunodeficiency (SID) is a hallmark of both malignancies predisposing patients to infections. Infection burden is high and can be both severe and recurrent [1, 11]. However, there is no clear consensus on optimal SID management in these patient groups [12]; current strategies focus on treating/preventing infections, using immunoglobulin‐replacement therapy, antimicrobial treatments and vaccination, but guidelines vary globally [1, 11]. A systematic review of randomised controlled trials in haematological malignancies found that, although 91% of studies report infection‐related data, methodologies vary and recurrent infections are often underreported, highlighting a need for more comprehensive studies in MM/CLL [13].

The study aimed to describe the epidemiology of infections and survival outcomes in Swedish patients with MM/CLL from 2010 to 2021 using real‐world data from national patient registries.

## 2 Materials and Methods

### 2.1 Study Design and Population

This is a non‐interventional, longitudinal, retrospective, population‐based study of patients diagnosed with MM/CLL in Sweden between January 2010 and December 2021. Data were obtained from four national administrative registries, providing complete nationwide registration due to governmental care: the National Patient Register (NPR) [14], the Prescribed Drug Register (PDR) [15], the Swedish Cancer Register [16] and the Cause of Death Register (CDR) [17]. Infections were identified using International Classification of Diseases (ICD) 10th Revision codes (Table S1) and captured in the NPR. Since Swedish healthcare is government funded, most healthcare visits and all prescribed drugs are registered and captured in these registries [18].

**FIGURE 1:**
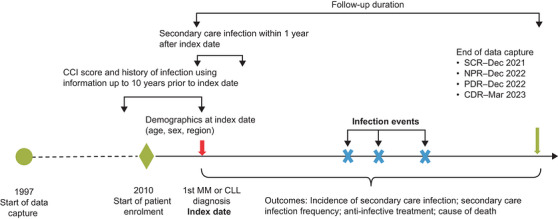
Outline of the Study design. CCI, Charlson Comorbidity Index; CDR=, ause of Death Register; CLL, Chronic lymphocytic leucemia; MM, Multiple myeloma; NPR, National Patient Register; PDR, Prescribed Drug Register; SCR, Swedish Cancer Register.

Patient data from the Swedish Cancer Register, linked to data captured in other registers, were included in the study if patients had received a first diagnosis of MM/CLL on or after January 2010, and aged ≥ 18 years at first diagnosis (Figure 1). Data on patients with MM (ICD‐O3 C90.0) and CLL (ICD‐O3 C91.1) were obtained from the Swedish Cancer Register between 2010 and 2021. Patients were followed from first diagnosis (index date) until end of follow‐up (date of death, end of data capture [March 2023 for mortality analyses] or emigration, whichever came first). Individual‐level data from the registries were linked to a unique personal identification number and merged into a single dataset, pseudonymised prior to use. Patients who emigrated or died ≤ 1 year from index date and those diagnosed with both MM and CLL were excluded from the study.

The study was reviewed and approved by the independent Swedish Ethical Review Board and conducted in accordance with the current Declaration of Helsinki and Good Pharmacoepidemiology Practices. Special attention was paid to data privacy protection, the European Union Data Protection Directive 95/46/EC.

### 2.2 Variables and Objectives

#### 2.2.1 Definition of Infections

Diagnosed and coded infections occurring in the secondary care setting, that is, in hospital or visits to specialised outpatient clinics, were included in this study. Infections were stratified as either “requiring hospitalisation” or “not requiring hospitalisation,” based on the recorded inpatient/outpatient register diagnosis. Infections requiring hospitalisation, where infection was the primary or secondary diagnosis, were further stratified by length of stay (≤ 3 or > 3 days). Infections not requiring hospitalisation included all specialist outpatient visits where infection was the primary or secondary diagnosis. Primary‐care visits to a medical doctor and other healthcare visits were excluded as they were not recorded in the NPR.

Since patients may have sought healthcare for the same infection on multiple occasions, diagnoses of the same infection within 4 weeks were considered a single event.

#### 2.2.2 Incidence of Infections

Patients were followed from diagnosis to first infection, to calculate accumulated person‐years. The formula used for calculating the incidence rate (IR) of infections is in Box S1.

#### 2.2.3 Incidence and Types of Recurrent Infections

All infections during follow‐up were identified and analysed as recurrent events. Patients were followed from diagnosis to migration, death or end of follow‐up, whichever came first, to calculate accumulated person‐years. The formula used for calculation of the IR of recurrent infection events is shown in Box S1. Infections were stratified by severity and type. Numbers and proportions of each type were calculated. The IR calculation formula used per infection type is shown in Box S1. These data were presented per person‐year and per 100 person‐years among patients.

#### 2.2.4 Predictors of Infection

Fine–Gray competing risk regression was used to identify infection predictors, accounting for competing mortality (death from non‐infection causes). Predictors were first evaluated in univariate Fine–Gray models, followed by multivariable analysis with stepwise selection (*p* < 0.05). The primary outcome was time to first documented infection, defined as time from haematological malignancy diagnosis to the first ICD‐10–coded infection. Patients without infection were censored at end of follow‐up.

Predictors included age at MM/CLL diagnosis, sex, Charlson Comorbidity Score, region of residency, prior infection history, prophylactic treatment and MM/CLL diagnosis year. A sensitivity analysis using a Cox‐proportional hazards model, treating death as a censoring event, was conducted to assess consistency across analytical approaches.

#### 2.2.5 Anti‐Infective Treatment

Types of anti‐infective treatment dispensed were determined by Anatomical Therapeutic Chemical codes (Table S2). These included antibiotics, antimycobacterials, antimycotics, antivirals, immunoglobulin and vaccines. All prescribed drugs are registered. The formula used for IR calculation for dispensations of anti‐infective treatment is shown in Box S1. The number and proportion of different types of anti‐infective treatment (excluding prophylaxis) used in the first anti‐infective treatment dispensation were calculated. The number and proportion of patients who received certain antibiotics often used prophylactically were calculated as a proxy for patients who received prophylactic anti‐infective treatment. Where necessary, data for subgroups of similar antibiotics were combined. Prophylactic use of antibiotics was defined as continuous use for ≥ 3 months.

#### 2.2.6 Overall Survival and Causes of Death

Overall survival (OS) was assessed as time from first MM/CLL diagnosis to death from any cause, including infection‐related death, and plotted using Kaplan–Meier modelling. Patients who did not die before the end of data capture, or were lost to follow‐up, were censored.

Causes of death were categorised into infection type; CLL, MM or concomitant cancers/secondary malignancies; other causes. In the Cause of Death Register, the underlying cause of death was defined as the disease/condition that initiated the events leading to death. The percentage of these patients was calculated separately for those with MM and CLL. The Cause of Death Register defines contributing causes as potentially exacerbating the underlying cause of death or contributing to overall decline in health, but not the primary cause (multiple causes may be reported) [19]. In patients for whom MM/CLL was the underlying cause of death, contributing causes were reported.

Infection‐related survival was analysed by using infection‐related cause of death as the event and death due to other causes as the competing event, while the non‐infection‐related survival used death due to other causes as the event, and infection‐related cause of death as the competing event.

### 2.3 Statistical Analysis

Continuous variables were summarised by sample size (*n*), mean and standard deviation (SD), median (Q1/Q3), range (minimum/maximum). Categorical variables were summarised using sample size (*n*), count and percentage by category. Descriptive statistics were used to analyse patient demographics: age, sex, Charlson Comorbidity Index (CCI) scores. Kaplan–Meier methodology was used to assess time to first infection and to first anti‐infective treatment. Reversed Kaplan–Meier curves were used for data presentation. The IR of infections was calculated as number of infections over accumulated person‐years and reported as number/person‐year. Patients were censored if an infection diagnosis was not received between index date–end of study, were lost to follow‐up, or died before diagnosis. Univariate/multivariable models in the Fine–Gray analyses were fitted using stepwise selection (*p* < 0.05). Cox‐proportional hazard models were fitted using the same stepwise selection procedure. All were performed using SAS v9.4. Log‐Rank and Wilcoxon significance tests were performed to assess OS differences between patients with/without infection. The Log‐Rank test provides equal weight to differences across the follow‐up period; the Wilcoxon test was more sensitive to differences occurring in the early follow‐up period.

## 3 Results

### 3.1 Patient Characteristics

A total of 15,766 patients were included in the study (MM: 8532; CLL: 7234) and all were followed up for a median (range) 3.3 (0–13)/4.8 (0–13) years, respectively. Most were male (MM: 57%; CLL: 62%), with a median (range) age of 71 (19–100) years (MM) and 71 (23–102) years (CLL) at diagnosis (Table 1). Mean (SD) CCI scores were 4.2 (2.2) (MM) and 3.5 (2.0) (CLL), respectively. The proportion of patients with a high (≥ 5) CCI was 37% (MM) and 25% (CLL). An infection history ≤ 10 years prior to diagnosis was recorded for 36% (MM)/30% (CLL) of patients, respectively.

**TABLE 1:** Patient characteristics.

Characteristics	MM	CLL
*N*	8532	7234
Sex, *n* (%)		
Male	4901 (57.4)	4500 (62.2)
Female	3631 (42.6)	2734 (37.8)
CCI category, *n* (%)		
Low (0–2)	2601 (30.5)	3094 (42.8)
Moderate (3–4)	2739 (32.1)	2328 (32.2)
High (≥ 5)	3192 (37.4)	1811 (25.0)
CCI, mean (SD)	4.19 (2.2)	3.54 (2.0)
History of infection up to 10 years prior to diagnosis, *n* (%)		
No	5434 (63.7)	5029 (69.5)
Yes	3098 (36.3)	2205 (30.5)
Patients with infection up to 1 year after diagnosis, *n* (%)		
No	5198 (60.9)	6056 (83.7)
Yes	3334 (39.1)	1178 (16.3)
Age, median (Q1, Q3)	71.0 (64.0, 78.0)	71.0 (64.0, 78.0)
Follow‐up duration, median (Q1, Q3)	3.3 (1.5, 5.6)	4.8 (2.5, 7.6)

Abbreviations: CCI, Charlson Comorbidity Index; CLL, chronic lymphatic leukaemia; MM, multiple myeloma; Q, quartile; SD, standard deviation.

### 3.2 Incidence of Infections

**FIGURE 2:**
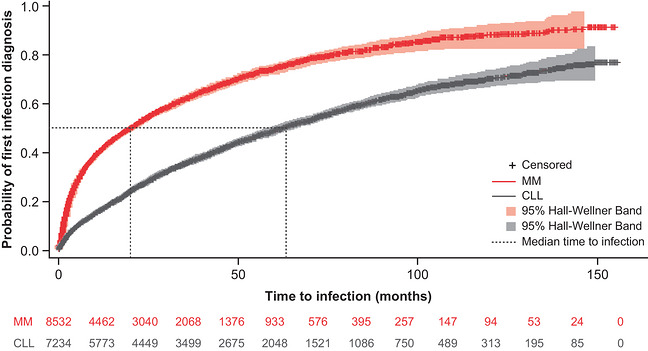
Time to first infection in patients with MM and CLL.

During follow‐up, 5618 (66%) patients with MM had at ≥ 1 infection (IR: 0.3), most leading to hospitalisation for > 3 days (IR: 0.2). For CLL, 3618 (50%) had ≥ 1 infection (IR: 0.1), with most treated in specialist‐based outpatient care (IR: 0.1). Median time from diagnosis to first infection was 1.7 (MM) and 5.2 (CLL) years (Figure 2).

### 3.3 Incidence and Types of Recurrent Infections

Patients with MM and CLL had 21,765 (IR: 0.7/person‐year) and 13,510 (IR 0.4/person‐year) infections, respectively, during follow‐up (Table 2). Of these, 30% (MM) and 36% (CLL) were treated in specialist outpatient care. For those with inpatient infections, most (50% [MM]; 45% [CLL]) required > 3 days of inpatient care.

**TABLE 2:** Number and IR of bacterial or viral infections by type in patients with MM or CLL.

	Patients with MM (*n* = 8532)			Patients with CLL (*n* = 7234)		
	Number of events (%)	IR per person‐year	IR per 100 person‐years	Number of events (%)	IR per person‐year	IR per 100 person‐years
**Any infection**	21,765	0.7	65.9	13,510	0.4	35.7
**By severity of infection**						
Inpatient infection						
≤ 3 days	4464 (20.5)	0.1	13.5	2469 (18.3)	0.1	7.5
> 3 days	10,801 (49.6)	0.3	32.7	6128 (45.4)	0.2	18.6
Outpatient infection	6500 (29.9)	0.2	19.7	4913 (36.4)	0.2	14.9
**Bacterial infections—any**	12,122 (55.7)	0.4	36.7	7609 (56.3)	0.2	20.1
Pneumonia	4581 (21.1)	0.1	13.9	2872 (21.3)	0.1	7.6
Urinary tract infections	1772 (8.1)	0.1	5.4	1338 (9.9)	0.0	3.5
Bacteraemia/sepsis	1732 (8.0)	0.1	5.2	775 (5.6)	0.0	2.0
Bacteraemia	744 (3.4)	0.0	2.3	351 (2.6)	0.0	0.9
Erysipelas	375 (1.7)	0.0	1.1	363 (2.7)	0.0	1.0
SIRS	389 (1.8)	0.0	1.2	227 (1.7)	0.0	0.6
Cellulitis	140 (0.6)	0.0	0.4	107 (0.8)	0.0	0.3
Endocarditis	149 (0.7)	0.0	0.5	62 (0.5)	0.0	0.2
Osteomyelitis	121 (0.6)	0.0	0.4	64 (0.5)	0.0	0.0
Meningitis	7 (0.0)	0.0	0.0	6 (0.0)	0.0	0.0
Pyelonephritis	> 5	N/A	N/A	> 5	N/A	N/A
**Viral infections—any**	2817 (12.9)	0.1	8.5	1767 (13.1)	0.1	4.7
Herpes zoster	554 (2.6)	0.0	1.7	476 (3.5)	0.0	1.3
Herpes simplex	157 (0.7)	0.0	0.5	142 (1.1)	0.0	0.4
Hepatitis B	118 (0.5)	0.0	0.4	104 (0.8)	0.0	0.3
Influenza	161 (0.7)	0.0	0.5	92 (0.7)	0.0	0.2
Hepatitis C	110 (0.5)	0.0	0.3	59 (0.4)	0.0	0.2
**Other infections—any**	6826 (31.4)	0.2	20.7	4134 (30.6)	0.1	10.9

Abbreviations: CLL, chronic lymphatic leukaemia; IR, incidence rate; MM, multiple myeloma; N/A, not applicable; SIRS, systemic inflammatory response syndrome.

Across all patients, compared with viral, bacterial infections were reported more frequently. Other (unspecified) infections were found in ∼31% of patients with MM/CLL; this comprised both viral and bacterial infections, including respiratory, abdominal, skin and other infections. The distribution of infection types was similar in both MM and CLL; pneumonia (21%) was the most common specified infection (Table 2). Herpes zoster was the most common specified viral infection and was more frequent in CLL (3.5%) than MM (2.6%), but remained less common than the unspecified infection group, which includes other viral infections such as respiratory infections.

### 3.4 Predictors of Infection

In multivariable Fine–Gray analyses, similar predictors of infection risk were identified in both cohorts. In MM, older age and female sex were significantly associated with lower risk (sub‐hazard ratio [SHR]: 0.985 [*p* < 0.0001], 0.924 [*p* = 0.0003]), while higher comorbidity burden remained a significant predictor of infection (moderate CCI SHR: 1.289; high CCI SHR: 1.574 [*p* < 0.0001 for both]) (Table 3). Prior infection and prophylactic treatment (versus no prior infection or prophylactic treatment, respectively) were significantly associated with increased infection risk (SHR: 1.183 [*p* < 0.0001] and 1.171 [*p* = 0.011]); more recent diagnosis was significantly associated with lower infection risk (SHR: 0.822 [*p* < 0.0001]). Regional differences were present, with modestly lower risk observed in Skåne, Västra Götalandsregionen and Uppsala/Örebro (SHR: 0.890 [*p* = 0.014], 0.912 [*p* = 0.045], 0.901 [*p* = 0.011], respectively) compared with Stockholm.

**TABLE 3:** Predictors of infection in patients with MM and CLL based on Fine–Gray analysis.

Variable	*p*‐value	Sub‐hazard ratio	Low CI	Upper CI
**Univariate analysis: MM**				
**Age**	<0.0001	0.988	0.986	0.990
**Sex** (ref = male)	<0.0001	0.898	0.852	0.947
**Charlson Comorbidity Index Score** (ref = low)				
Moderate	<0.0001	1.244	1.162	1.333
High	<0.0001	1.542	1.445	1.646
**Prior infection history** (ref = no secondary infections prior to MM/CLL diagnosis)	<0.0001	1.201	1.138	1.268
**Prophylactic treatment** (ref = prophylaxis not used prior to MM/CLL diagnosis)	0.0010	1.225	1.086	1.381
**Region** (ref = Stockholm)				
Skåne	0.0120	0.888	0.809	0.974
VGR	0.0070	0.883	0.807	0.967
Uppsala/Örebro	0.0010	0.875	0.807	0.948
Norrland	0.4270	0.961	0.872	1.059
Sydöstra	0.5620	0.976	0.900	1.059
**MM diagnosis year** (ref = 2010–2012)				
2013–2015	0.4620	0.974	0.909	1.044
2016–2018	0.0600	0.935	0.872	1.003
2019–2021	<0.0001	0.773	0.715	0.836
**Multivariable analysis: MM**				
**Age**	<0.0001	0.985	0.982	0.987
**Sex** (ref = male)	0.003	0.924	0.876	0.974
**Charlson Comorbidity Index Score** (low)				
Moderate	<0.0001	1.289	1.203	1.383
High	<0.0001	1.574	1.473	1.683
**Prior infection history** (ref = no secondary infections prior to MM/CLL diagnosis)	<0.0001	1.183	1.120	1.250
**Prophylactic treatment** (ref = prophylaxis not used prior to MM/CLL diagnosis)	0.011	1.171	1.037	1.321
**Region** (ref = Stockholm)				
Skåne	0.014	0.890	0.811	0.976
VGR	0.045	0.912	0.833	0.998
Uppsala/Örebro	0.011	0.901	0.832	0.976
Norrland	0.764	0.985	0.895	1.085
Sydöstra	0.863	0.993	0.915	1.077
**MM diagnosis year** (ref = 2010–2012)				
2013–2015	0.498	0.976	0.911	1.047
2016–2018	0.505	0.977	0.911	1.047
2019–2021	<0.0001	0.822	0.760	0.890
**Univariate analysis: CLL**				
**Age**	<0.0001	1.010	1.007	1.014
**Sex** (ref = male)	<0.0001	0.846	0.791	0.906
**Charlson Comorbidity Index Score** (ref = low)				
Moderate	<0.0001	1.629	1.504	1.765
High	<0.0001	2.182	2.013	2.365
**Prior infection history** (ref = no secondary infections prior to MM/CLL diagnosis)	<0.0001	1.640	1.531	1.756
**Prophylactic treatment** (ref = prophylaxis not used prior to MM/CLL diagnosis)	0.001	1.317	1.119	1.550
**Region** (ref = Stockholm)				
Skåne	0.222	0.928	0.822	1.047
VGR	0.051	0.898	0.806	1.001
Uppsala/Örebro	0.028	0.893	0.808	0.988
Norrland	0.032	0.868	0.763	0.988
Sydöstra	0.521	0.967	0.873	1.071
**MM diagnosis year** (ref = 2010–2012)				
2013–2015	<0.0001	0.863	0.796	0.936
2016–2018	<0.0001	0.796	0.730	0.867
2019–2021	<0.0001	0.661	0.593	0.737
**Multivariable analysis: CLL**				
**Age**	0.033	1.004	1.000	1.007
**Sex** (ref = male)	<0.0001	0.869	0.812	0.932
**Charlson Comorbidity Index Score** (ref = low)				
Moderate	<0.0001	1.512	1.393	1.642
High	<0.0001	1.904	1.748	2.075
**Prior infection history** (ref = no secondary infections prior to MM/CLL diagnosis)	<0.0001	1.498	1.397	1.606
**Prophylactic treatment** (ref = prophylaxis not used prior to MM/CLL diagnosis)	0.008	1.248	1.058	1.471
**Region** (ref = Stockholm)				
Skåne	0.092	0.900	0.795	1.017
VGR	0.134	0.920	0.825	1.026
Uppsala/Örebro	0.013	0.880	0.796	0.973
Norrland	0.047	0.877	0.771	0.998
Sydöstra	0.301	0.947	0.855	1.050
**MM diagnosis year** (ref = 2010–2012)				
2013–2015	0.002	0.879	0.810	0.954
2016–2018	<0.0001	0.832	0.762	0.909
2019–2021	<0.0001	0.705	0.630	0.788

Abbreviations: CI, confidence interval; CLL, chronic lymphatic leukaemia; MM, multiple myeloma; VGR, Västra Götalandsregionen.

In CLL multivariable analyses, age was associated with a modestly increased risk (SHR: 1.004 [*p* = 0.033]), while female sex was associated with lower infection risk (SHR: 0.869 [*p* < 0.0001]) (Table 3). Comorbidity burden remained strongly associated with infection risk (moderate CCI SHR: 1.512; high CCI SHR: 1.904 [*p* < 0.0001 for both]), as did prior infection and prophylactic treatment (SHR: 1.498 [*p* < 0.0001], 1.248 [*p* = 0.008]). Lower risk was observed in Uppsala/Örebro (SHR: 0.880 [*p* = 0.013) and Norrland (SHR: 0.877 [*p* = 0.047]); more recent diagnosis was associated with lower infection risk (SHR: 0.705 [*p* < 0.0001]).

Cox‐proportional hazards models results were consistent with the Fine–Gray findings; similar associations observed for key predictors, including comorbidity, prior infection, prophylaxis, sex and diagnosis year (Table S3).

### 3.5 Non‐Prophylactic Anti‐Infective Treatment Patterns

**FIGURE 3:**
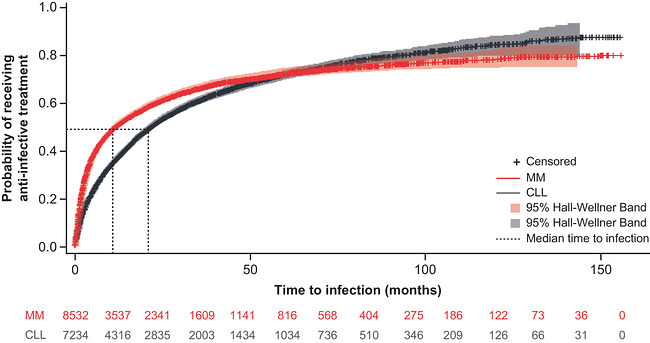
Time to first anti‐infective treatment, excluding prophylaxis, in patients with MM or CLL.

Throughout the study, 5370 (63%) patients with MM and 4918 (68%) with CLL had ≥ 1 anti‐infective treatment dispensed (excluding prophylaxis) (Table 4). Median time from diagnosis to first anti‐infective treatment was 11 (MM) and 21 (CLL) months (Figure 3). The most frequently dispensed anti‐infective treatment was antibiotics (MM: 55%; CLL: 64%), followed by antimycotics (MM: 16%; CLL: 9%) (Table 4).

**TABLE 4:** Patients with MM or CLL receiving ≥ 1 anti‐infective treatment during the study, by treatment type.

	Patients with MM (*n* = 8532)	Patients with CLL (*n* = 7234)
**IR of dispensations of anti‐infective treatment (per person‐year)**	4.8	1.9
**Any anti‐infective, *n* (%)**	5370 (62.9)	4918 (68.0)
Antibiotics	4695 (55.0)	4641 (64.2)
Antimycobacterials	60 (0.7)	26 (0.4)
Antimycotics	1331 (15.6)	661 (9.1)
Antivirals	56 (0.7)	80 (1.1)
Immunoglobulin	121 (1.4)	88 (1.2)
Vaccines	199 (2.3)	265 (3.7)
None of the above	3162 (37.1)	2316 (32.0)

Abbreviations: CLL, chronic lymphatic leukaemia; IR, incidence rate; MM, multiple myeloma.

### 3.6 Prophylactic Anti‐Infective Treatment Patterns

The proportion of patients using anti‐infective treatments as prophylaxis (continuous specific antibiotics/antivirals use for ≥ 3 months) was 76% (MM) and 39% (CLL) (Table S4). Among those treated with ≥ 1 anti‐infective treatment (6448: MM; 2818: CLL), 96% (*n* = 6199) and 77% (*n* = 2175), respectively, received acyclovir and/or valaciclovir.

### 3.7 OS and Infection‐Related Death

**FIGURE 4:**
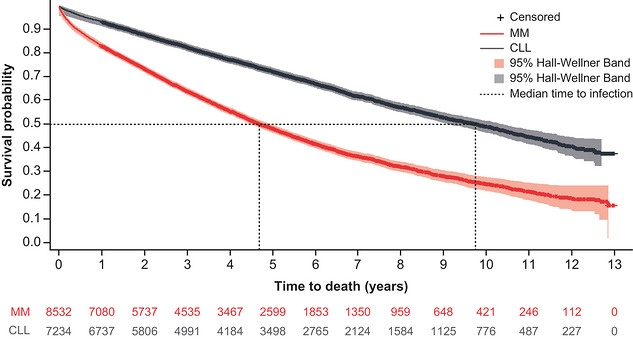
Overall survival.

Median OS (95% confidence interval [CI]) was 4.7 (4.6–4.9) years (MM) and 9.7 (9.3–10.2) years (CLL) (Figure 4). During the study, 4822 patients with MM, and 2571 with CLL, died. The most common underlying cause of death was the respective malignancy (67% and 33%, respectively) (Table 5). Infection (any) was the main cause of death in 235/4822 (4.9%) and the main/contributing cause of death for 1251/4822 (26%) patients with MM. Among deceased patients with CLL, infection (any) was the underlying cause of death in 249/2571 (9.7%) and the underlying/contributing cause of death for 835/2571 (32%). Among deceased patients with CLL, 69% died from other causes as a main/contributing cause. Similarly in patients with MM, this was 78%. These were mostly related to cardiovascular causes.

**TABLE 5:** Cause of death in study population.

	Underlying cause of death		Underlying and/or contributing cause of death		Contributing cause of death	
Cause of death, *n* (%)	MM	CLL	MM	CLL	MM	CLL
***N***	4822 (100)	2571 (100)	4822 (100)	2571 (100)	4822 (100)	2571 (100)
**Infection—any type**	235 (4.9)	249 (9.7)	1251 (25.9)	835 (32.5)	1115 (23.1)	678 (26.4)
Bacterial	59 (1.2)	50 (1.9)	578 (12.0)	379 (14.7)	537 (11.1)	343 (13.3)
Viral	35 (0.7)	24 (0.9)	84 (1.7)	70 (2.7)	77 (1.6)	54 (2.1)
Other	51 (1.1)	48 (1.9)	681 (14.1)	437 (17.0)	633 (13.1)	392 (15.3)
COVID‐19	90 (1.9)	127 (4.9)	109 (2.3)	145 (5.6)	19 (0.4)	18 (0.7)
**MM/CLL**	3247 (67.3)	852 (33.1)	4143 (85.9)	1639 (63.8)	899 (18.6)	789 (30.7)
**Other cancers**	317 (6.6)	541 (21.0)	652 (13.5)	778 (30.3)	482 (10.0)	517 (20.1)
**Other causes of death**	1023 (21.2)	929 (36.1)	3332 (69.1)	2004 (78.0)	3190 (66.2)	1867 (72.6)

Abbreviations: CLL, chronic lymphatic leukaemia; COVID‐19, coronavirus disease 2019; MM, multiple myeloma.

**FIGURE 5:**
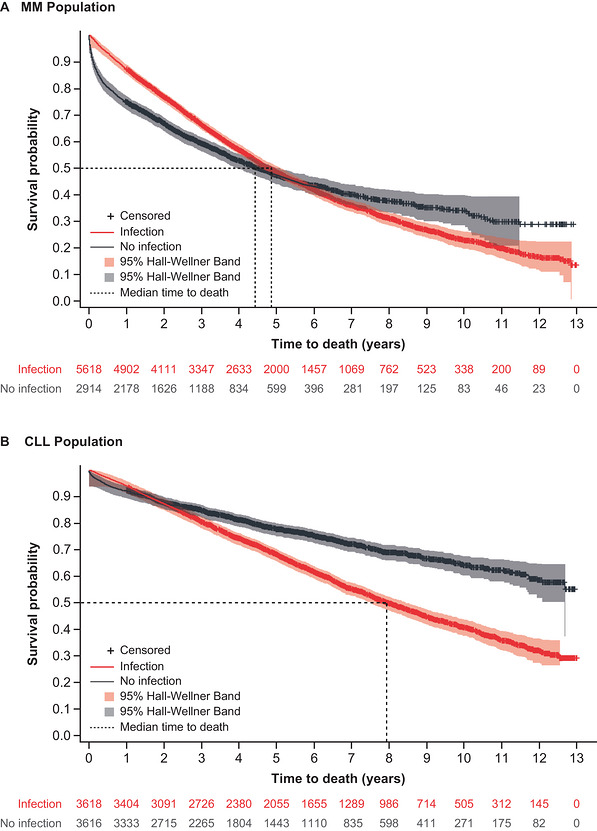
Infection‐related survival in patients with MM (A) and CLL (B).

Patients with MM and CLL who experienced infection during follow‐up had worse median OS (MM: 4.47 years; 95% CI: 4.11–4.85) (CLL: 7.97 years; 95% CI: 7.61–8.34) compared with those without infection (MM: 4.87 years; 95% CI: 4.65–5.05) (CLL: not reached 50% of survival probability) (Figure 5). OS differed significantly between patients with/without infection in both cohorts. In the MM cohort, a larger Wilcoxon than log‐rank statistic (*χ* ^2^ = 90.17 [*p* < 0.0001] versus 9.35 [*p* = 0.0022]) indicated that survival differences were most pronounced in the follow‐up period. In the CLL cohort, while both statistical tests were significant, the log‐rank statistic was larger than the Wilcoxon (*χ* ^2^ = 159.74 versus 66.96 [*p* < 0.0001 for both]), suggesting that survival differences were sustained throughout the follow‐up period (Table S5).

## 4 Discussion

In this large, nationwide, 12‐year study of > 15,000 patients with MM and CLL, we observed a high incidence of patients experiencing ≥ 1 infection (MM: 66%; CLL: 50%). Infections occurred early in the disease course, with a median time to first infection of 2 (MM)/5 (CLL) years; ∼33% had an infection before their confirmed diagnosis. Although the underlying malignancy was the most common cause of death, infections were a substantial contributing factor, emphasising the need for improved infection‐risk stratification, targeted prevention strategies and improved treatment for haematological malignancies.

This study confirmed that infections remain a significant burden for these patients, likely attributed to the severe immunodeficiency. It is known that chemoimmunotherapy for haematological malignancies is strongly linked to defects in humoural and cellular immunity [20]. In the past decade, there has been a shift in MM treatment, with anti‐CD38 monoclonal antibodies and expanded use of triplet/quadruplet regimens [21, 22]. In Sweden, daratumumab was approved for use in patients with MM in 2016 [23] with combination treatment recommended in 2020 for those newly diagnosed [24] by the Swedish New Therapies Council; however, lenalidomide is now commonly used across treatment lines and regimens [25]. Studies have shown that patients treated with anti‐CD38 monoclonal antibodies have increased risk of severe infections, including bacterial pneumonia and sepsis [26]. Thus, reliance on daratumumab‐based regimens in Sweden may explain the high infection rates in MM observed. At the start of the study period, chemoimmunotherapy (e.g. chemotherapy plus anti‐CD20 antibodies) was commonly used as first‐line treatment for CLL. However, treatment practices have evolved over time, with increasing adoption of targeted therapies, including BTK inhibitors (BTKi) and venetoclax‐based regimens [27]. In Sweden, BTKi use has gradually increased since 2017, with broader uptake of B‐cell receptor pathway inhibitor‐based therapies observed from 2020 onwards [28].

Although targeted approaches to the treatment of MM and CLL have led to improvements in patient survival [22, 29], this comes at the cost of increasing the risk of secondary health complications, including infections as patients age [29]. Additional factors predicting an increase in CLL infections are the presence of comorbid chronic obstructive pulmonary disease, the use of two or more prior therapies, age, unmutated immunoglobulin heavy chain variable region, more advanced Binet stage and presence of hypogammaglobulinaemia [20, 30]. In MM, patients who had undergone autologous stem cell transplantation had a higher infection load 2 years after diagnosis [31]. In our study, higher comorbidity burden, prior infection history and prophylactic treatment were consistently associated with increased infection risk, whilst age, female sex and more recent diagnosis reduced risk. These factors reinforce the need for targeted screening at diagnosis to enable proactive clinical management.

Our findings align with prior population‐based studies reporting an elevated risk of pneumonia and sepsis in MM/CLL [2, 32, 33]. A study using Swedish Myeloma Registry data between 2008 and 2021 demonstrated that patients with MM had ∼5‐fold increased infection risk compared to matched controls [32]. A high infection rate was observed in this population, with many receiving immunomodulatory drugs, proteasome inhibitors or monoclonal antibodies. Similar to our study, most were bacterial, leading to septicaemia and pneumonia [32]. A population‐based study of patients with CLL from the Swedish Cancer Registry, between 1994 and 2013, demonstrated a > 15‐fold increased risk of inpatient opportunistic infection compared to matched controls. The highest IRs were noted for pneumocystis pneumonia, herpes zoster, *Candida, Pseudomonas* and aspergillosis [33]. For both malignancy types, the most common specified infections were pneumonia, urinary tract infections and bacteriaemia/sepsis. Our data show persistently high infection rates despite evolving treatment strategies, necessitating ongoing improvements in infection prevention and care.

Our study provides important real‐world evidence on the timing and patterns of infections. We found that infections occurred early after an MM diagnosis, with a median time to first infection of ∼2 years. The time to first infection was longer with CLL, occurring ∼5 years after diagnosis. This may reflect differing treatment demands at diagnosis. Notably, nearly one third of patients had experienced an infection within 10 years prior to diagnosis. During the first year after diagnosis, MM infection rates remained high (40%); CLL 16%; this aligns with previous observations. A population‐based study of mantle cell lymphoma demonstrated high infection rates in the 4 years preceding diagnosis, persisting ≤ 8 years afterwards [34]. It has been demonstrated that patients with B‐cell malignancies, including MM/CLL, are at increased infection risk ≤ 15 years before diagnosis, indicating that deterioration of the immune system often develops before malignancy [35].

Most patients in our study received ≥ 1 non‐prophylactic anti‐infective agent, usually antibiotics. For infection treatment with MM, European guidelines recommend immediate broad‐spectrum antibiotics [21]. Among patients with a particularly high risk of infection, and those treated with lenalidomide/pomalidomide, antimicrobial prophylaxis is recommended [21]. Patients with CLL and a high risk of developing complications or recurrent infections are recommended antibiotic/antiviral prophylaxis [27]. Around 1% of patients in our study received immunoglobulins. Guidelines state that immunoglobulin replacement therapy should be reserved for those experiencing severe hypogammaglobulinaemia and repeated/severe infections [27]; however, use may be underreported in this study, as immunoglobulins ordered and administered by hospitals or clinics, rather than prescribed, may not be captured in prescribing records.

There was a significant OS difference between patients with/without infection, and death rates where infection was a contributing factor: 26% (MM) and 32% (CLL)—consistent with previous studies. Between 2008 and 2021, a Swedish registry study of > 8500 patients with MM reported that infection contributed to 32%/27% of all deaths within 2 months/1 year post diagnosis, respectively [32]. Another Swedish study, which followed patients up to 2007, demonstrated that 22% of MM deaths were infection related [2]. Studies of CLL infection‐related deaths have shown similar results. In a Danish population study, 29% of CLL deaths were infection related [36]. A Japanese study reported that CLL infections contributed to 30% of deaths [37]. These underscore the burden of infection‐related mortality despite use of modern therapies.

Unspecified MM/CLL infections, both bacterial and viral, were found in ∼31% of patients, with viral respiratory infections the most frequent; this is consistent with findings that these are among the most common in patients with haematological malignancies [38]. However, they were not consistently specified in the ICD coding, likely contributing to the high proportion of unspecified infections observed. Among infections that were explicitly classified, herpes zoster was the most common viral infection.

Our study has notable strengths. Our IR calculations for recurring infections (MM: 66; CLL: 36, per 100 person‐years) correspond to estimates of 66% (MM) and 36% (CLL) of patients experiencing ≥ 1 infection per year. The actual percentage of patients experiencing an infection may be higher, when accounting for infections diagnosed in the primary‐care setting. We followed methodological guidance on the use of Swedish national administrative health registers [39] to identify a total MM/CLL Swedish population reliably. The use of limited selection bias and data sources enabled us to follow study outcomes longitudinally. In addition, registries are nationwide and government funded, ensuring complete capture of infections (2001 onwards) and prescribed drug use (2005 onwards).

Some limitations should be considered. Patients comprised a secondary‐care population, meaning no infections diagnosed in a primary‐care setting were identified. However, as all patients had an MM/CLL diagnosis, they were expected to visit secondary care regularly, limiting potential impact on study results. This study was not designed to capture information on disease characteristics or other clinical variables that may contextualise the population's findings. We did not collect data on MM/CLL‐specific treatments, and our inclusion criteria did not distinguish treated from untreated patients. Furthermore, comparison of the infection data with that from a healthy control group was not possible, but would have provided useful insights. This study utilised Swedish data; therefore, different healthcare structures could impact how patients are affected by infections elsewhere.

This study confirms that infections represent a significant burden, occurring frequently and early in the disease course. The results emphasise the importance of infection management in haematological malignancies and warrant further investigation into their impact on patient outcomes and healthcare resource utilisation.

## Author Contributions

I.G., L.T., J.F., M.N.S., Q.Y., A.D. and S.Y.K. contributed to study design and conception and data interpretation. Q.Y. and N.S. contributed to the data analysis. All authors equally contributed to drafting, review and editing of the manuscript, and revised and approved the final version.

## Funding

This study was funded by Takeda, Sweden. Medical writing support was provided by Kate Bradford, PhD, of Parexel, under the direction of the authors, and funded by Takeda, Sweden.

## Ethics Statement

The study was reviewed and approved by the independent Swedish Ethical Review Board (Etikprövningsnämnderna) and was conducted in accordance with the current version of the Declaration of Helsinki and Good Pharmacoepidemiology Practices. Special attention was paid to data privacy protection, the European Union Data Protection Directive 95/46/EC.

## Consent

The study makes use of routinely collected data that was de‐personalised before release; therefore, informed consent from individual participants was not required.

## Conflicts of Interest

Ingrid Glimelius has received financial support to the department from Takeda, participated in educational symposium with financial support to the department provided by Lilly and participated in educational symposia organised by AbbVie, Johnson & Johnson and Kite‐Gilead. Love Tätting has attended scientific expert group meetings and received honoraria from Amgen, Bristol Myers Squibb, Pfizer and Sanofi and received honoraria from Takeda for participation on this trial's Executive Committee. Jonatan Freilich, M. Natalia Stelmaszuk and Qian Yang were employed by Parexel during the conduct of the study. Parexel is a global contract research organization that provides clinical research and development services to pharmaceutical and biotechnology companies. Anna Deleskog is an employee of Takeda Pharma AB, Sweden. Sigurður Y. Kristinsson has received research grants from Amgen and Celgene/Bristol Myers Squibb.

## Supporting information



Supporting Information: jha270363‐sup‐0001‐SuppMat

## Data Availability

Data were obtained from four Swedish national administrative registries owned by the National Board of Health and Welfare (NBHW, Socialstyrelsen). Ethical approval was obtained. Agreement between the NBHW and researchers only allows the use of data by individuals approved in the data extraction agreement.
