# Supplementary material for: Infection Incidence and Survival in Patients With Multiple Myeloma and Chronic Lymphocytic Leukaemia: A Growing Concern in the Era of Modern Therapies
Source: EJHaem. 2026 Jul 31;7(4):e70363. doi: 10.1002/jha2.70363 (PMC13426477; doi:10.1002/jha2.70363)
Supplement: Supplementary file 1 — Supporting Information: jha270363‐sup‐0001‐SuppMat [file JHA2-7-e70363-s001.docx]

**Supplementary Appendix**

**Infection Incidence and Survival in Patients with Multiple Myeloma and Chronic Lymphocytic Leukaemia: A Growing Concern in the Era of Modern Therapies**

**Authors:**

**Ingrid Glimelius│ Love Tätting │ Jonatan Freilich│ M. Natalia Stelmaszuk │ Qian Yang│ Anna Deleskog │ Sigurður Y. Kristinsson**

**Supplementary Appendix**

**SUPPLEMENTARY TABLE 1 │** ICD-10 codes of infections.

| Category of infection | ICD-10 codes used in the study |
| --- | --- |
| Bacterial | A00, A01, A02, A03, A04, A05, A15, A16.1, A16.2, A16.3, A16.5, A16.7, A16.8, A16.9, A17, A18, A19, A20, A21, A22, A23, A24, A25, A26, A27, A28, A30, A31, A32, A33, A34, A35, A36, A37, A38, A39, A40, A41, A42, A43, A44, A46, A48, A49, A50-51, A52, A53, A54, A55, A56, A57, A58, A65, A66, A67, A68, A69, A70, A71, A74, A75, A77, A78, A79, B90, B92, B95, B96, G00, G01, G06, G07, G08, I33, I39, J01, J13, J14, J15, J36, J38, J39.0, J39.1, H70, J86, K10.2, K12.2, K35, K61, K63.0, K65, K67, K75.0, K80.0, K80.1, K80.3, K80.4, K81.0, K81.9, K83.0, M46.2–M46.5, M86, N10, N30, N34, N39.0, N45, L00, L01, L02, L03, L08.0B, L08.8, L08.8A, L08.8B, L08.8C, L08.8W, L08.9, M00, T81.4, T82.6, T82.7, T83.5, T83.6, T84.7, T85.7, R57.2, R65.0, R65.1 |
| Viral | A08, A60, A80, A81, A82, A83, A84, A85, A86, A87, A88, A89, A92, A93, A94, A95, A96, A97, A98, A99, B00, B01, B02, B03, B04, B05, B06, B07, B08, B09, B15, B16, B17, B18, B19, B20, B21, B22, B23, B24, B25, B26, B27, B30, B33, B34, B91, B97, J00, J09, J10, J11, J12 |
| Other | A06, A07, A09, A16.0, A59, A63, A64, B35, B36, B37, B38, B39, B40, B41, B42, B43, B44, B45, B46, B47, B48, B49, B50, B51, B52, B53, B54, B55, B56, B57, B58, B60, B64, B65, B66, B67, B68, B69, B70, B71, B72, B73, B74, B75, B76, B77, B78, B79, B80, B81, B82, B83, B85, B86, B87, B88, B89, B94, B98, B99, D80.1, D80.6, G02, G03, G04, G05, H00, H44.0, H05.0, I30, I32, I40, I41, J02, J03, J04, J05, J06, J16, J17, J18, J20, J21, J22, M60.0, M65.0–M65.1, M71.0–M71.1, N11, N12, N13.6, N15.9, N16.0, N41, N48.2, N49, N51, N70, N71, N72, N73, N74, N75, N76, N77, L04, L05, L08.0X, M01, T80.2, T84.5, T84.6, T88.0 |

Abbreviations: ICD-10, International Statistical Classification of Diseases and Related Health Problems, 10th revision; SIRS, systemic inflammatory response syndrome.

**SUPPLEMENTARY TABLE 2 │** Anti-infective ATC codes.

| Treatment category | ATC codes |
| --- | --- |
| Antibacterials for systemic use | J01, A07AA09, A07AA12 |
| Antimycotics for systemic use | J02 |
| Antimycobacterials | J04, A07AA02 |
| Antivirals for systemic use | J05 |
| Immune sera and immunoglobulins | J06 |
| Vaccines | J07 |
| Treatments used as prophylaxis |  |
| Acyclovir | J05AB01 |
| Valaciclovir | J05AB11 |
| Bactrim, Bactrim Forte, Eusaprim, Eusaprim Forte | J01EE01 |
| Fluconazole | J02AC01 |
| Posaconazole | J02AC04 |

Abbreviation: ATC, Anatomic Treatment Classification.

**SUPPLEMENTARY TABLE 3 │** Predictors of infection in patients with MM or CLL based on Cox-proportional hazard models.

| **UNIVARIATE ANALYSIS: MM** | | | | |
| --- | --- | --- | --- | --- |
| **Variable** | ***p*-value** | **Sub-hazard ratio** | **Low CI** | **Upper CI** |
| **Age** | 0.6854 | 1.001 | 0.998 | 1.003 |
| **Sex** (ref=male) | < 0.0001 | 0.898 | 0.852 | 0.948 |
| **Charlson Comorbidity Index Score** (ref=low) |  |  |  |  |
| Moderate | < 0.0001 | 1.391 | 1.298 | 1.491 |
| High | < 0.0001 | 1.760 | 1.648 | 1.879 |
| **Prior infection history** (ref=no secondary infections prior to MM/CLL diagnosis) | < 0.0001 | 1.386 | 1.314 | 1.463 |
| **Prophylactic treatment** (ref=prophylaxis not used prior to MM/CLL diagnosis) | 0.0002 | 1.267 | 1.12 | 1.434 |
| **Region** (ref=Stockholm) |  |  |  |  |
| Skåne | 0.0481 | 0.910 | 0.829 | 0.999 |
| VGR | 0.1103 | 0.928 | 0.847 | 1.017 |
| Uppsala/Örebro | 0.0417 | 0.919 | 0.847 | 0.997 |
| Norrland | 0.8223 | 1.011 | 0.917 | 1.115 |
| Sydöstra | 0.4933 | 1.029 | 0.948 | 1.116 |
| **MM diagnosis year** (ref=2010–2012) |  |  |  |  |
| **2013–2015** | 0.3159 | 0.964 | 0.896 | 1.036 |
| **2016–2018** | 0.0741 | 0.937 | 0.871 | 1.006 |
| **2019–2021** | < 0.0001 | 0.839 | 0.775 | 0.909 |
| **MULTIVARIABLE ANALYSIS: MM** | | | | |
| **Variable** | ***p*-value** | **Sub-hazard ratio** | **Low CI** | **Upper CI** |
| **Age** | 0.0157 | 0.997 | 0.994 | 0.999 |
| **Sex** (ref=male) | 0.0007 | 0.912 | 0.864 | 0.962 |
| **Charlson Comorbidity Index Score** (ref=low) |  |  |  |  |
| Moderate | < 0.0001 | 1.357 | 1.265 | 1.464 |
| High | < 0.0001 | 1.685 | 1.575 | 1.822 |
| **Prior infection history**  (ref=no secondary infections prior to MM/CLL diagnosis) | < 0.0001 | 1.317 | 1.247 | 1.388 |
| **Prophylactic Treatment** (ref=prophylaxis not used prior to MM/CLL diagnosis) | 0.0057 | 1.192 | 1.052 | 1.346 |
| **Region** (ref=Stockholm) |  |  |  |  |
| Skåne | 0.0452 | 0.909 | 0.828 | 0.999 |
| VGR | 0.2598 | 0.949 | 0.865 | 1.042 |
| Uppsala/Örebro | 0.1127 | 0.936 | 0.863 | 1.017 |
| Norrland | 0.7320 | 1.017 | 0.922 | 1.125 |
| Sydöstra | 0.4561 | 1.032 | 0.951 | 1.119 |
| **MM diagnosis year** (ref=2010–2012) |  |  |  |  |
| **2013–2015** | 0.3943 | 0.969 | 0.901 | 1.036 |
| **2016–2018** | 0.5693 | 0.979 | 0.911 | 1.006 |
| **2019–2021** | 0.0061 | 0.893 | 0.824 | 0.909 |
| **UNIVARIATE ANALYSIS: CLL** | | | | |
| **Variable** | ***p*-value** | **Sub-hazard ratio** | **Low CI** | **Upper CI** |
| **Age** | < 0.0001 | 1.024 | 1.021 | 1.028 |
| **Sex** (ref=male) | < 0.0001 | 0.823 | 0.769 | 0.881 |
| **Charlson Comorbidity Index Score** (ref=low) |  |  |  |  |
| Moderate | < 0.0001 | 1.707 | 1.574 | 1.85 |
| High | < 0.0001 | 2.555 | 2.356 | 2.771 |
| **Prior Infection History**  (ref=no secondary infections prior to MM/CLL diagnosis) | < 0.0001 | 1.806 | 1.687 | 1.933 |
| **Prophylactic Treatment**  (ref=prophylaxis not used prior to MM/CLL diagnosis) | 0.0012 | 1.303 | 1.11 | 1.53 |
| **Region** (ref=Stockholm) |  |  |  |  |
| Skåne | 0.7311 | 1.021 | 0.905 | 1.153 |
| VGR | 0.4837 | 0.962 | 0.862 | 1.073 |
| Uppsala/Örebro | 0.2617 | 0.943 | 0.852 | 1.044 |
| Norrland | 0.1796 | 0.914 | 0.801 | 1.042 |
| Sydöstra | 0.5701 | 1.03 | 0.929 | 1.143 |
| **MM diagnosis year** (ref=2010–2012) |  |  |  |  |
| **2013–2015** | 0.0082 | 0.892 | 0.82 | 0.971 |
| **2016–2018** | 0.0005 | 0.854 | 0.78 | 0.934 |
| **2019–2021** | < 0.0001 | 0.739 | 0.66 | 0.826 |
| **MULTIVARIABLE ANALYSIS: CLL** | | | | |
| **Variable** | ***p*-value** | **Sub-hazard ratio** | **Low CI** | **Upper CI** |
| **Age** | < 0.0001 | 1.017 | 1.013 | 1.02 |
| **Sex** (ref=male) | < 0.0001 | 0.829 | 0.774 | 0.889 |
| **Charlson Comorbidity Index Score** (ref=low) |  |  |  |  |
| Moderate | < 0.0001 | 1.498 | 1.379 | 1.628 |
| High | < 0.0001 | 2.058 | 1.887 | 2.244 |
| **Prior infection history**  (ref=no secondary infections prior to MM/CLL diagnosis) | < 0.0001 | 1.616 | 1.508 | 1.732 |
| **Prophylactic treatment**  (ref=prophylaxis not used prior to MM/CLL diagnosis) | 0.0109 | 1.234 | 1.05 | 1.45 |
| **Region** (ref=Stockholm) |  |  |  |  |
| Skåne | 0.7015 | 0.977 | 0.865 | 1.103 |
| VGR | 0.6831 | 0.977 | 0.876 | 1.091 |
| Uppsala/Örebro | 0.1335 | 0.925 | 0.835 | 1.024 |
| Norrland | 0.1527 | 0.908 | 0.796 | 1.036 |
| Sydöstra | 0.9216 | 0.995 | 0.897 | 1.104 |
| **MM diagnosis year** (ref=2010–2012) |  |  |  |  |
| **2013–2015** | 0.0430 | 0.916 | 0.842 | 0.997 |
| **2016–2018** | 0.0395 | 0.909 | 0.83 | 0.995 |
| **2019–2021** | 0.0009 | 0.823 | 0.735 | 0.923 |

VGR, Västra Götalandsregionen.

**SUPPLEMENTARY TABLE 4 │** Patients with MM or CLL receiving anti-infective treatments as prophylaxis.

|  | Patients with MM  (*n* = 8532) | Patients with CLL (*n* = 7234) |
| --- | --- | --- |
| ≥ 1 anti-infective, *n* (%) | 6448 (75.6) | 2818 (39.0) |
| Acyclovir and/or valaciclovir | 6199 (72.7) | 2175 (30.1) |
| Bactrim and/or Bactrim Forte and/or Eusaprim and/or Eusaprim Forte | 3796 (44.5) | 1859 (25.7) |
| Fluconazole AND/OR posaconazole | 1872 (21.9) | 656 (9.1) |

Abbreviations: CLL, chronic lymphatic leukaemia; MM, multiple myeloma.

**SUPPLEMENTARY TABLE 5 │** Significance tests for overall survival in patients with and without infections.

|  | Test | Chi-Square | DF | *p-*value |
| --- | --- | --- | --- | --- |
| MM | Log-rank | 9.3518 | 1 | 0.0022 |
|  | Wilcoxon | 90.1692 | 1 | < 0.0001 |
| CLL | Log-rank | 159.7372 | 1 | < 0.0001 |
|  | Wilcoxon | 66.9630 | 1 | < 0.0001 |

Abbreviations: CLL, chronic lymphatic leukaemia; DF, degrees of freedom; MM, multiple myeloma

**SUPPLEMENTARY BOX 1 │** Formulae for calculations.

- 1. **Incidence rate (IR) of infections**
  $IR of first infection=\frac{Total number of firstinfection events}{Accumulated person-years}$
  2. **IR of recurrent infection events**

*IR of recurrent infection*

- 1. **IRs of each type of infection**

*IR of specific type of infection*

- 1. **IR of dispensations of anti-infective treatment:**

*IR of anti-infective treatment*$= \frac{\sum dispensations of anti-infective treatment}{\sum follow-up duration}$
